# A nomogram for predicting hemorrhagic shock in pediatric patients with multiple trauma

**DOI:** 10.1038/s41598-024-62376-6

**Published:** 2024-06-10

**Authors:** Nan Lin, Jingyi Jin, Sisi Yang, Xiaohui Zhong, Hang Zhang, Yichao Ren, Linhua Tan, Hongzhen Xu, Daqing Ma, Jinfa Tou, Qiang Shu, Dengming Lai

**Affiliations:** 1 Department of Neonatal Surgery, Children’s Hospital, Zhejiang University School of Medicine, National Clinical Research Center for Child Health, Hangzhou, 310052 China; 2 Department of Thoracic and Cardiovascular Surgery, Children’s Hospital, Zhejiang University School of Medicine, National Clinical Research Center for Child Health, Hangzhou, 310052 China; 3 Department of Surgical Intensive Care Unit, Children’s Hospital, Zhejiang University School of Medicine, National Clinical Research Center for Child Health, 310052 Hangzhou, China; 4 Perioperative and Systems Medicine Laboratory, National Clinical Research Center for Child Health, Children’s Hospital, Zhejiang University School of Medicine, 310052 Hangzhou, China; 5 Division of Anaesthetics, Pain Medicine & Intensive Care, Department of Surgery & Cancer, Faculty of Medicine, Imperial College London, Chelsea & Westminster Hospital, London, UK

**Keywords:** Pediatric, Multiple trauma, Hemorrhagic shock, Prediction nomogram, Diseases, Medical research, Risk factors

## Abstract

The timely detection and management of hemorrhagic shock hold paramount importance in clinical practice. This study was designed to establish a nomogram that may facilitate early identification of hemorrhagic shock in pediatric patients with multiple-trauma. A retrospective study was conducted utilizing a cohort comprising 325 pediatric patients diagnosed with multiple-trauma, who received treatment at the Children's Hospital, Zhejiang University School of Medicine, Zhejiang, China. For external validation, an additional cohort of 144 patients from a children's hospital in Taizhou was included. The model's predictor selection was optimized through the application of the Least Absolute  Shrinkage and Selection Operator (LASSO) regression. Subsequently, a prediction nomogram was constructed using multivariable logistic regression analysis. The performance and clinical utility of the developed model were comprehensively assessed utilizing various statistical metrics, including Harrell's Concordance Index (C-index), receiver operating characteristic (ROC) curve analysis, calibration curve analysis, and decision curve analysis (DCA). Multivariate logistic regression analysis identified systolic blood pressure (ΔSBP), platelet count, activated partial thromboplastin time (APTT), and injury severity score (ISS) as independent predictors for hemorrhagic shock. The nomogram constructed using these predictors demonstrated robust predictive capabilities, as evidenced by an impressive area under the curve (AUC) value of 0.963. The model's goodness-of-fit was assessed using the Hosmer–Lemeshow test (χ^2^ = 10.023, *P* = 0.209). Furthermore, decision curve analysis revealed significantly improved net benefits with the model. External validation further confirmed the reliability of the proposed predictive nomogram. This study successfully developed a nomogram for predicting the occurrence of hemorrhagic shock in pediatric patients with multiple trauma. This nomogram may serve as an accurate and effective tool for timely and efficient management of children with multiple trauma.

## Introduction

Accident injuries continue to be a leading cause of mortality in pediatric and adolescent population with the age range from 1 to 18 years old globally^1,2.^ Among these injuries, multiple trauma, often resulting from vehicular accidents and falls, stands out with the highest mortality rate^3^. Approximately 50% of mortalities due to hemorrhagic shock occurred within the initial six-hour post-traumatic period^4^. Pediatric patients experiencing multiple trauma undergo a complicated and multifaceted cascade of pathological changes including significant blood loss, high neuroendocrine stress response and hemodynamic instability^5,6.^ Due to the relatively small blood volume in pediatric patients, injuries can lead to substantial blood loss at the onset of injury, resulting in rapid hypovolemia towards hemorrhagic shock^7^. In addition, the physiological response to trauma in children significantly differs from that in adults. In children, normal arterial blood pressure can be maintained through heart rate and systemic vascular resistance changes, even with approximately 25% to 30% blood loss^8^ and hence, once hemorrhagic shock occurs, it is often fatal. Therefore, timely diagnosis and management of traumatic hemorrhagic shock are far more than important for children with multiple trauma^9^.

The novel bimodal time-to-trauma death model with two critical timeframes for mortality: "immediate/within 60 min" and "within 4–48 h" was introduced for surgical and/or critical care trauma management and this model gradually replaces the conventional three-peak time-to-trauma death distribution^10^. The first 60 min following trauma have been identified as a crucial window for preclinical trauma management. Thus, there is an urgent need for the development of risk prediction models that can forecast the occurrence of hemorrhagic shock upon admission in children presented with multiple trauma.

Nevertheless, due to the complex nature of multiple trauma in pediatric patients, identifying individuals at risk of hemorrhagic shock remains a challenge. Children often present with shock symptoms such as anxiety, excitement, or irritability that differ from those in adults. Consequently, physicians can use physiological parameters and clinical signs to assess the presence of shock together with base excess (BE), lactate (Lac), and admission blood glucose (Glu) as early predictors in adult populations^11–13^ which are not reliable in pediatrics. Owe to this, our objective is to develop a predictive nomogram that incorporates a range of clinical indicators within the initial 60 min following a child's traumatic injury. This risk prediction model for hemorrhagic shock is expected to provide robust decision support to healthcare professionals. It, therefore, can be used to plan treatment strategies, including fluid resuscitation, blood transfusion, and surgical interventions, based on the individual patient's risk profile. Such an approach is poised to enhance the timeliness and effectiveness of treatment, ultimately reducing mortality rates.

## Methods

### Study design and population

The study protocol was approved by the Ethics Committee of Children's Hospital, Zhejiang University School of Medicine and Taizhou Women and Children’s Hospital with a waiver for informed consent (2023-IRB-0045-P-01). Procedures were followed in accordance with the ethical standards of the responsible committee on human experimentation and the Helsinki Declaration of 1975. The retrospective study was designed to analyze the data from 325 traumatic young patients treated at the Children's Hospital, Zhejiang University School of Medicine, from January 1, 2019, to April 30, 2023. For external validation, an additional cohort of 144 patients admitted to a children's hospital in Taizhou during the same period of time as above was included for analysis. Inclusion criteria comprised patients diagnosed with multiple trauma. Exclusion criteria encompassed: (a) patients who arrived already in hemorrhagic shock and (b) patients who passed away before reaching the hospital.

### Data collection

The data collector retrieved medical records specifically from patients diagnosed with multiple injuries among the broader pool of trauma patients. The initial recorded value in the electronic medical record was adopted for all measurements. In addressing missing data, cases with multiple missing data required for this study were excluded. For instances where only individual data points were missing, we retained the cases within the study population and employed multiple imputation techniques to fill in the missing values. In selecting the variables, the study consulted relevant literature and, considering clinical expertise and experience, identified 19 indicators for inclusion in the analysis. The data collected for this study encompassed general demographics such as age and gender, along with specific information regarding the cause of injury and the organs affected. Physiological parameters measured included systolic blood pressure (SBP), diastolic blood pressure (DBP), and body temperature. Additionally, a comprehensive set of laboratory data was gathered, which included measurements of hemoglobin (Hb), white blood cells (WBC), platelets (PLT), activated partial thromboplastin time (APTT), prothrombin time (PT), pH level, lactate (Lac), base excess (BE), hematocrit (HCT), and blood glucose (Glu). The severity of the injuries was quantified using the Injury Severity Score (ISS), and the extent of any resulting coma was assessed with the Glasgow Coma Scale (GCS).

### Definitions

In this study, multiple trauma was defined as two or more anatomical injuries simultaneously or successively under the action of a single mechanical injury, one of which, even if present alone, may be life-threatening^14^. Additionally, hemorrhagic shock, as characterized in this study, represents a pathophysiological condition induced by trauma. It entails substantial blood loss from the body, thereby diminishing the effective circulating blood volume, resulting in inadequate tissue perfusion, disruptions in cellular metabolism, and compromised organ function^15^. In recognition of the variability in standard blood pressure values across pediatric age groups, this study adopted a methodology that utilized adjusted systolic and diastolic pressures. Specifically, the observed systolic blood pressures in the pediatric cohort were compared to established age-specific norms by subtracting the standard values^16^ from the observed values, resulting in a measurement referred to as ΔSBP (Delta-SBP), were analyzed to ensure accurate and age-appropriate assessments. Subsequently, these adjusted values, including ΔSBP, were incorporated into the analyses, providing a tailored and precise dataset for further evaluation.

### Statistical analysis

The sample size was calculated using SPSS 26.0 and R 4.0.5. Descriptive statistics were performed on categorical data by numbers and percentages, and chi-squared tests or Fisher exact probability methods were used for comparison between groups. For continuous data, the normal distribution was expressed as mean ± standard deviation (SD), and the t-test was used for comparison between groups, while the skewed distribution was expressed as median (M) and interquartile range (IQR), and Mann–Whitney U test was then used for comparison. The study utilized LASSO regression to pinpoint potential predictive features within the patient population. Subsequently, the optimal value of λ was determined by minimizing the CV function, aiming to identify the most fitting model within one standard error of the minimum value. Backward stepwise multivariable logistic regression analysis was then conducted, using the features selected in the LASSO model to pinpoint significant predictors that could be utilised in creating a nomogram. The Hosmer–Lemeshow test was used to assess the model's goodness of fit. The model's predictive accuracy and conformity were assessed with the area under the ROC curve (AUC) and the calibration curve. The net benefit of the model for patients was done with the decision curve analysis (DCA). Discrimination and calibration were evaluated by bootstrapping with 1000 resamples to ensure accuracy. External validity was assessed through computing model's predictive accuracy in the validation cohort using the C-index and calibration plots. The validation cohort underwent the model to obtain individual risk scores. To categorize patients in the validation cohort as low-risk and high-risk groups, optimal threshold values from the training cohort were applied and their predictions were compared with the actual occurrence of shock. To evaluate the utility of the model, the sensitivity and specificity using the cut-off points in both the training and validation groups were calculated.

## Results

### General characteristics

**Figure 1:**
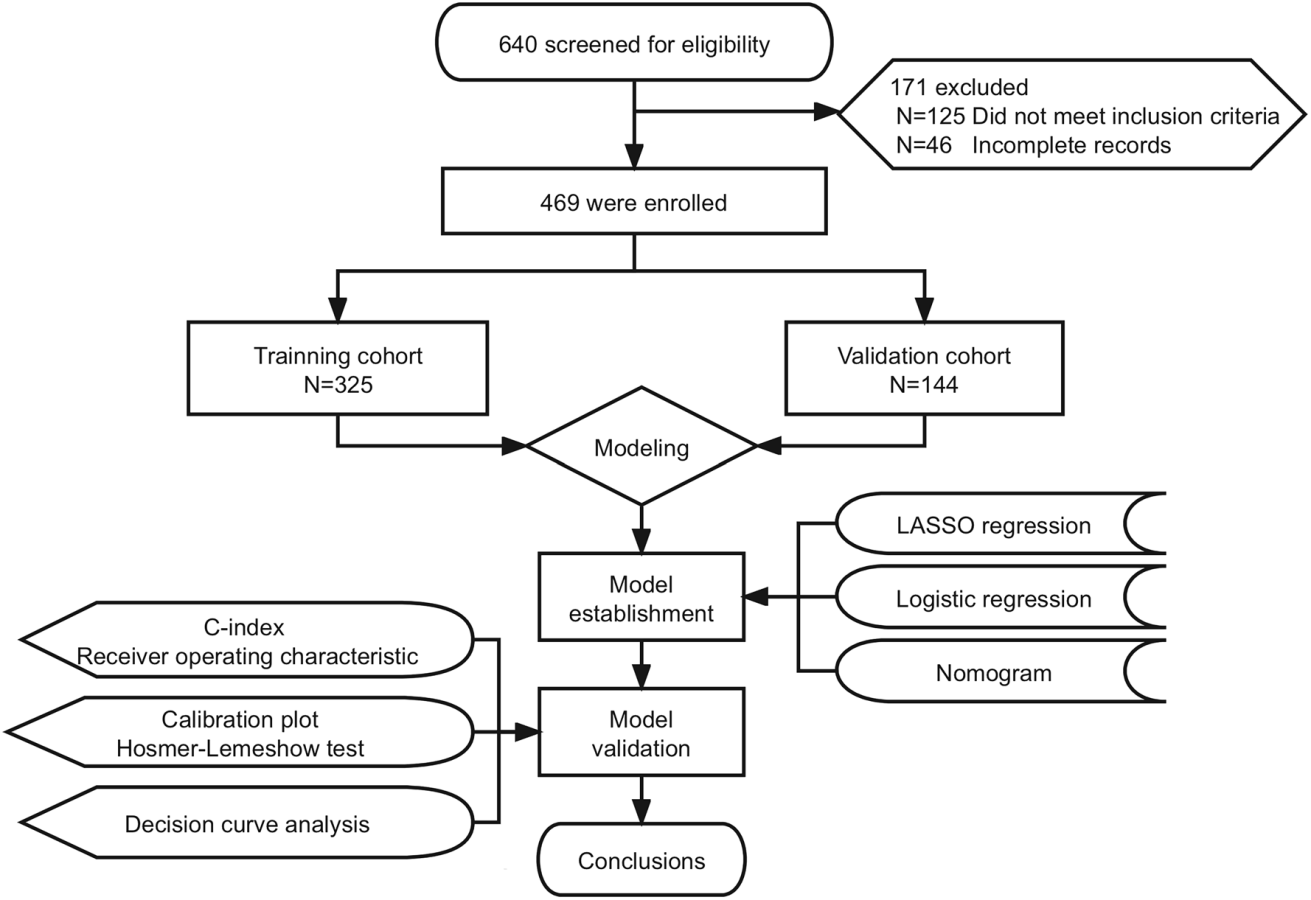
Flow chart for patient selection. C-index, concordance index; LASSO, least absolute shrinkage and selection operator.

Clinical information was gathered from 640 patients. 171 were excluded for not meeting the inclusion criteria while 46 (32 from training cohort and 14 from validation cohort) had incomplete records, specifically missing Glasgow score, APTT, PT, PLT, and ISS. Consequently, 469 patients were enrolled in this study; of those, 325 were training cohort and 144 for validation use (Fig. 1). The children’s age was from 2 to 13[4.75(2.80–7.80)] years, including 301 males (64.2%) and 168 females (35.8%). There were 224 cases (47.8%) of traffic injuries, 184 cases (39.2%) of falling injuries, and 61 cases (13.0%) of other causes. 371 patients, accounting for 79.10% of the total, were brought to the emergency department within 30 min of their injury. In total, 47 patients, constituting 10.0% of the overall cohort, experienced hemorrhagic shock. Specifically, within the training group, 34 patients (10.5%) manifested hemorrhagic shock, while in the validation group, 13 patients (9.0%) exhibited this condition. The baseline demographics of all patients in both the groups are presented in Table 1.

**Table 1:** Baseline characteristics of all patients in the training cohort and validation cohort.

Variables	Training cohort(n = 325) M (P25, P75)/N (%)	Validation cohort(n = 144) M (P25, P75)/N (%)	*P-*value*
Age (years)	4.67 (2.75, 7.75)	5.00 (2.90, 7.80)	0.459
Gender			0.767
Female	115 (35.4)	53 (36.8)	
Male	210 (64.6)	91 (63.2)	
Cause of injury			0.066
Traffic injury	146 (44.9)	78 (54.2)	
Falling injury	130 (40.0)	54 (37.5)	
Others	49 (15.1)	12 ( 8.3)	
Organ injury			0.096
Yes	151 (46.5)	55 (38.2)	
No	174 (53.5)	89 (61.8)	
ΔSBP (mmHg)	15.00 (6.00, 28.00)	17.00 (8.00, 26.00)	0.659
ΔDBP (mmHg)	3.00 (-6.00, 13.00)	4.00 (-4.00, 14.00)	0.733
Body temperature (℃)	37.20 (36.60, 37.70)	37.00 (36.70, 37.50)	0.151
Hb (g/L)	103.00 (88.00, 116.00)	105.00 (90.00, 118.00)	0.325
WBC (× 10^9^/L)	13.42 (9.86, 18.74)	13.45 (9.12, 19.18)	0.770
PLT (× 10^9^/L)	241.00 (176.00, 305.00)	251.50 (189.00, 315.00)	0.368
APTT (s)	27.10 (24.90, 30.10)	27.60 (24.70, 32.20)	0.328
PT (s)	12.90 (11.80, 16.20)	12.90 (11.70, 15.40)	0.486
pH	7.39 (7.34, 7.42)	7.39 (7.35, 7.42)	0.61
Lac (mmol/L)	2.00 (1.30, 3.30)	2.10 (1.37, 3.40)	0.678
BE (mmol/L)	-2.90 (-5.00, -1.10)	-3.20 (-4.90, -1.48)	0.377
HCT	32.70 (28.00, 36.60)	33.50 (29.17, 36.40)	0.488
Glu (mmol/L)	7.30 (6.20, 9.20)	7.50 (6.37, 9.30)	0.376
GCS	13.00 (8.00, 13.00)	12.00 (7.00, 13.00)	0.116
ISS	19.00 (10.00, 29.00)	18.00 (12.00, 23.00)	0.082

*P-value compare the characteristics in the training and validation cohorts using Wilcoxon Mann–Whitney test or exact Fisher test depending on whether the variable is continuous or categorical.

Δ*SBP* = Delta-systolic blood pressure, Δ*DBP* = Delta-diastolic blood pressure, *Hb* = hemoglobin, *WBC* = white blood cells, *PLT* = platelets, *PTT* = activated partial thrombin time, *PT* = prothrombin time, *Lac* = lactate, *BE* = base excess, *HCT* = hematocrit, *Glu* = blood glucose, *ISS* = injury severity score, *GCS* = glasgow coma scale.

### Screening for predictive factors

**Figure 2:**
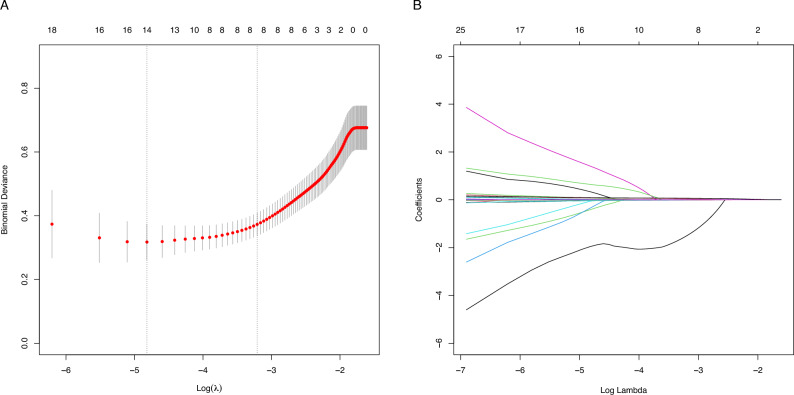
Predictor selection using LASSO regression analysis with tenfold cross-validation. (**A**) Selection of the tuning parameter (lambda) of the deviation in the LASSO regression based on the minimum criteria (left dotted line) and the 1-SE criteria (right dotted line). (**B**) A coefficient profile plot was generated against the log (lambda) sequence. In this study, the predictor was selected according to the 1-SE criteria (right dotted line), where 8 non-zero coefficients were selected. LASSO, least absolute shrinkage and selection operator; SE, standard error.

Of the 19 parameters collected from patients (Table 1), eight features were chosen based on non-zero coefficients calculated by LASSO logistic regression analysis (Fig. 2A and 2B). These selected parameters were ΔSBP, Hb, PLT, APTT, PT, pH, Lac, and ISS which were subsequently included in multivariate logistic regression analysis. A multivariate logistic regression analysis identified that ΔSBP, PLT, APTT, and ISS were independent factors predicting hemorrhagic shock (Table 2).

**Table 2:** Multivariate analysis of predictors selected by LASSO regression procedure in the training cohort.

Variables	Definition	Estimate OR (95% CI)	*P-*value
ΔSBP	Continuous, mmHg	0.956(0.921,0.992)	0.016
PLT	Continuous, × 10^9^/L	0.990(0.984,0.997)	0.005
APTT	Continuous, s	1.068(1.018,1.121)	0.008
ISS	Continuous	1.117(1.061,1.176)	< 0.001

Δ*SBP* = Delta-systolic blood pressure, *PLT* = platelets, *APTT* = activated partial thrombin time, *ISS* = injury severity score.

### Risk prediction nomogram development

**Figure 3:**
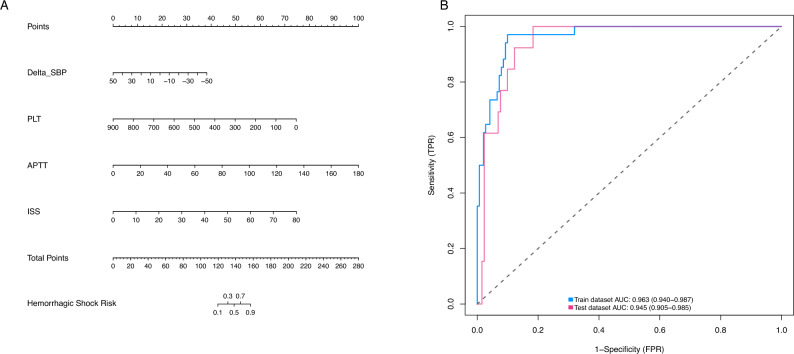
Nomogram and ROC curves for predicting the risk of hemorrhagic shock. (**A**) Nomogram for prediction of hemorrhagic shock. First, for each variable of a patient, a point was found on the highest rule, then all points were added and the total number of points was collected. Finally, the corresponding predicted probability of hemorrhagic shock was found on the lowest rule. (**B**) ROC curves. The blue curve shows the AUC of the training model and the red curve shows the AUC of the validation model. ROC, receiver operating characteristic curve; AUC, area under the curve.

The above independent predictors were then incorporated to develop a predictive nomogram (C-index = 0.963) (Fig. 3A). For each patient, higher total points indicated as a higher risk of hemorrhagic shock. In addition, the Hosmer–Lemeshow test demonstrated that the model was a good fit (χ^2^ = 10.023, *P* = 0.209).

### Risk prediction model validation

**Figure 4:**
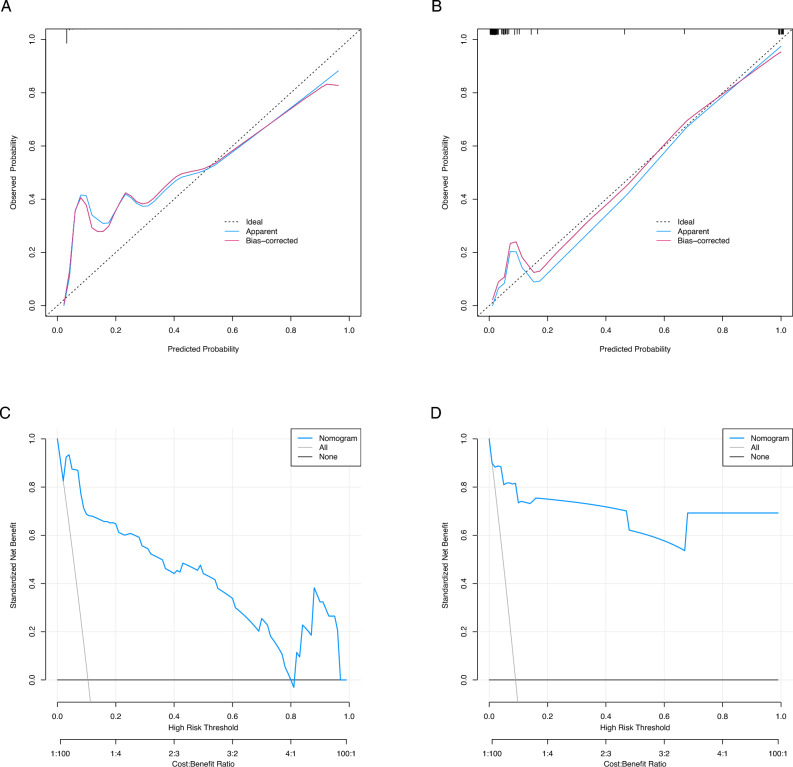
Calibration curves and decision curves of the hemorrhagic shock nomogram. (**A**) Calibration curves for the nomogram model in the training cohort; (**B**) Calibration curves for the nomogram model in the validation cohort; Notes: The x-axis represents the predicted hemorrhagic shock risk and the y-axis represents the actual observed hemorrhagic shock. The diagonal dotted line represents perfect prediction by an ideal model. The solid line reflects the performance of the nomogram; a closer fit to the dashed line indicates a better prediction. (**C**) Decision curves for the nomogram model in the training cohort; (**D**) Decision curves for the nomogram model in the validation cohort. Notes: The blue line represents the hemorrhagic shock risk model; the thin line represents the predict-all-patients as hemorrhagic shock and the thick line represents the predict-none-patients as hemorrhagic shock.

In the training cohort, the AUC was 0.963 (Fig. 3B). The maximum Youden index, a metric of diagnostic performance, reached 0.871, identifying an optimal threshold value of 0.102. This threshold exhibited a sensitivity of 0.971 and a specificity of 0.900. Moreover, the calibration plot exhibited a notable concordance with the ideal diagonal line (Fig. 4A).

For external validation, a distinct cohort comprising 144 patients was utilized to evaluate the nomogram's performance. The AUC for the validation cohort was determined to be 0.945 (Fig. 3B), accompanied by an impressive sensitivity of 0.892 and a specificity of 0.931. This affirmed the accuracy of the nomogram in outcome prediction. Additionally, the model demonstrated consistency, and the calibration curve for the validation cohort was closely paralleled with the ideal diagonal line (Fig. 4B).

Furthermore, Decision Curve Analysis (DCA) illuminated that the optimal utility of the nomogram, for predicting the risk of hemorrhagic shock, spanned probability thresholds between 3 and 96% in the training set, and between 2 and 99% in the validation set (Fig. 4C and D). Within this range, the net benefit was found to be comparable with several overlapping regions, as per the nomogram's predictions. Moreover, in the validation group, a calculation based on the best cut-off value derived from the training group identified 11 individuals at a high risk of hemorrhagic shock. Notably, this subset yielded 13 actual cases of hemorrhagic shock, thereby underscoring the model's accuracy, which reached 97.22%.

## Discussion

Our study found that ΔSBP, PLT, APTT, and ISS were independently wield significant influence over the probability of hemorrhagic shock manifestation in pediatric patients with multiple injuries. Hypotension serves as a crucial clinical marker for extensive blood loss within trauma patients^17^. It is noteworthy that children possess a substantial inherent physiological reserve against shock, hence hypotensive episodes are seldom observed until blood loss escalates to the extent of 45%^17^. This intrinsic resilience exhibited by children allows for the transient tolerance of diminished blood pressure levels before manifesting overt signs of hypotension. Consequently, the early detection of hemorrhagic shock in pediatric patients presents a notable challenge, given their initial compensatory mechanisms to mitigate blood loss.

This study confirms that PLT count reliably predicts hemorrhagic shock. Children in shock had an average PLT count of 141, contrasting starkly with the non-shock group's average of 256. A previous study showed that a 10*10^9/L rise in platelets reduces acute lung injury risk by 29% and 28-day mortality by 11%^18^. Trauma-Induced Coagulopathy affects 25–33% of those with hemorrhagic trauma. Thrombocytopenia predicts poor post-trauma outcomes, including increased transfusions, worsening brain injury, and higher mortality rates^19^. Even with seemingly normal platelet counts, many trauma patients show impaired platelet function right after the injury, a trend seen consistently in animal models of injury and hemorrhagic shock^20^. Therefore, monitoring and assessing PLT counts are crucial in clinically detecting and managing hemorrhagic shock promptly.

This study emphasizes the predictive importance of APTT in foreseeing the emergence of hemorrhagic shock. The continuous activation of the coagulation process early in severe trauma creates a highly coagulated and viscous environment in the patient's circulation, significantly increasing the chances of intravascular coagulation and blood clotting incidents. As elucidated in previous investigations^21^, the severity of trauma-related hemorrhage is positively correlated with the likelihood of concomitant coagulation and fibrinolytic dysregulation. Notably, the study by Zhang et al. underscores the substantial clinical value of APTT measurement in trauma patients for prognostic assessment and monitoring disease progression^22^.

The ISS is widely used in trauma assessment, but this study focuses on its particular relevance in predicting hemorrhagic shock. The data shows a stark contrast: the hemorrhagic shock group averaged an ISS score of 41, while non-shocked children averaged 17. Recent investigations suggested that, for children, an ISS score exceeding 25 may be a more fitting threshold to delineate severe injuries^23^. Furthermore, when scrutinizing the necessity for full trauma team activation in the pediatric cohort, an ISS threshold surpassing 23 is deemed as the optimal point of demarcation^23^. While the ISS typically predicts patient mortality, this study extends its use to early identification of hemorrhagic shock. This innovative approach aims to improve clinical decision-making by promptly recognizing shock using the ISS, ultimately reducing mortality and adverse outcomes.

In this study, a nomogram model has been established with four clinical predictors to facilitate risk prediction and early intervention. Internal validation of the model has confirmed its reliability. A higher composite score assigned to a child signifies an elevated risk of hemorrhagic shock in the context of multiple trauma patients. This visual predictive model equips healthcare practitioners with an uncomplicated, intuitive, and user-friendly instrument for the prompt detection of hemorrhagic shock. As per our model's recommendations, children with multiple trauma require expeditious assessment upon admission, with an emphasis on distinguishing those at high and low risk. Those identified as high-risk candidates should promptly receive intravenous access, crystalloid resuscitation, and subsequent blood product transfusions^24,25^. Naturally, children categorized as low risk should be subjected to follow-up observation and monitoring, remaining vigilant for any fluctuations in the predictive parameters. This model holds the potential to serve as a valuable tool for primary care hospitals to readily discern high-risk cases and administer appropriate interventions early, thus mitigating the prospect of overtreatment among low-risk patients to some extent.

## Limitations

While this study introduces a valuable predictive nomogram for hemorrhagic shock in pediatric trauma patients, it is constrained by several limitations that merit consideration. The study's geographic confinement to hospitals in Zhejiang, China, potentially restricts the extrapolation of findings to different regions or healthcare environments. Moreover, its retrospective design, reliant on the integrity of medical records, may be susceptible to biases stemming from incomplete or inaccurately recorded data. Furthermore, although the application of LASSO regression mitigates the risk of overfitting, it may inadvertently omit marginally significant but clinically relevant predictors. The lack of prehospital data and variability in initial medical care might compromise the accuracy of critical predictors such as ΔSBP and APTT. Additionally, the homogeneity in the ethnic composition of the study cohort may not adequately represent the diverse demographic profiles encountered in broader populations. These factors necessitate a prudent interpretation of the results and underscore the importance of expanding data collection in future research to refine the nomogram's relevance and precision across varied clinical settings.

## Conclusion

In conclusion, ΔSBP, PLT, APTT, and ISS were identified to be independent risk factors for hemorrhagic shock in children with multiple trauma. A nomogram with high predictive performance was constructed with these risk factors. Our findings may provide clinicians with a simple and intuitive tool, which is important for reducing mortality in children with severe trauma but warrants further validation before applied to clinical practice.

## Data Availability

The datasets used and/or analyzed during the current study available from the corresponding author on reasonable request.

## References

[1] Olaisen RH, Rossen LM, Warner M, Anderson RN (2019). Unintentional injury death rates in rural and urban areas: United States, 1999–2017. NCHS Data Brief..

[2] West BA, Rudd RA, Sauber-Schatz EK, Ballesteros MF (2021). Unintentional injury deaths in children and youth, 2010–2019. J. Saf. Res..

[3] Jelodar S, Jafari P, Yadollahi M (2014). Potential risk factors of death in multiple Trauma patients. Emerg (Tehran).

[4] Duque P, Mora L, Levy JH, Schochl H (2020). Pathophysiological response to Trauma-Induced coagulopathy: A comprehensive review. Anesth. Analg..

[5] Al Hanna R, Amatya B, Lizama LE, Galea MP, Khan F (2020). Multidisciplinary rehabilitation in persons with multiple trauma: A systematic review. J. Rehabil. Med..

[6] Pape HC, Moore EE, McKinley T, Sauaia A (2022). Pathophysiology in patients with polytrauma. Injury.

[7] Karam O, Russell RT, Stricker P (2018). Recommendations on RBC transfusion in critically Ill children with nonlife-threatening bleeding or hemorrhagic shock from the pediatric critical care transfusion and anemia expertise initiative. Pediatr. Crit. Care Med.

[8] Elsayed Y, Abdul Wahab MG (2022). A new physiologic-based integrated algorithm in the management of neonatal hemodynamic instability. Eur. J. Pediatr..

[9] Russell RT, Esparaz JR, Beckwith MA (2023). Pediatric traumatic hemorrhagic shock consensus conference recommendations. J. Trauma Acute Care Surg..

[10] Eriksson J, Nelson D, Holst A, Hellgren E, Friman O, Oldner A (2021). Temporal patterns of organ dysfunction after severe trauma. Crit. Care.

[11] Kreutziger J, Rafetseder A, Mathis S, Wenzel V, El Attal R, Schmid S (2015). Admission blood glucose predicted haemorrhagic shock in multiple trauma patients. Injury.

[12] Taherinia A, Saba G, Ebrahimi M (2021). Diagnostic value of intravenous oxygen saturation compared with arterial and venous base excess to predict hemorrhagic shock in multiple trauma patients. J. Family Med. Primary Care.

[13] Ozturk Ormeci G, Yigit O, Eray O (2022). Utility of ETCO2 to predict hemorrhagic shock in multiple trauma patients. Turk. J. Med. Sci..

[14] Hui Li DD (2023). Evolution of and controversy on definition of multiple trauma. Chin. J. Trauma.

[15] Standl T, Annecke T, Cascorbi I, Heller AR, Sabashnikov A, Teske W (2018). The nomenclature, definition and distinction of types of shock. Dtsch. Arztebl. Int..

[16] Flynn JT, Kaelber DC, Baker-Smith CM (2017). Clinical practice guideline for screening and management of high blood pressure in children and adolescents. Pediatrics.

[17] Ko Y, Kim JH, Hwang K, Lee J, Huh Y (2021). Comparison of base deficit and vital signs as criteria for hemorrhagic shock classification in children with Trauma. Yonsei Med. J..

[18] Wu M, Luan YY, Lu JF (2020). Platelet count as a new biomarker for acute kidney injury induced by hemorrhagic shock. Platelets.

[19] Kornblith LZ, Moore HB, Cohen MJ (2019). Trauma-induced coagulopathy: The past, present, and future. J. Thromb. Haemost..

[20] Schaub LJ, Moore HB, Cap AP, Glaser JJ, Moore EE, Sheppard FR (2017). Nonhuman primate model of polytraumatic hemorrhagic shock recapitulates early platelet dysfunction observed following severe injury in humans. J. Trauma Acute Care Surg.

[21] Delano MJ, Rizoli SB, Rhind SG (2015). Prehospital resuscitation of traumatic hemorrhagic shock with hypertonic solutions worsens hypocoagulation and hyperfibrinolysis. Shock.

[22] Zhang L, Lin M, Tang X, Tang Y (2022). Correlation between coagulation fibrinolysis function and outcomes during hospitalization in patients with severe traumatic hemorrhagic shock. Emerg. Med. Int..

[23] Chegondi M, Hernandez Rivera JF, Alkhoury F, Totapally BR (2023). The need for blood transfusion therapy is associated with increased mortality in children with traumatic brain injury. PLoS One.

[24] Deeb AP, Lu L, Guyette FX (2023). Optimal prehospital crystalloid resuscitation volume in trauma patients at risk for hemorrhagic shock. J. Am. Coll. Surg..

[25] Polites SF, Moody S, Williams RF (2020). Timing and volume of crystalloid and blood products in pediatric trauma: An Eastern Association for the Surgery of Trauma multicenter prospective observational study. J. Trauma Acute Care Surg..

